# Transdiagnostic cognitive behaviour therapy for adolescents with an eating disorder who are not underweight

**DOI:** 10.1016/j.brat.2015.07.014

**Published:** 2015-10

**Authors:** Riccardo Dalle Grave, Simona Calugi, Massimiliano Sartirana, Christopher G. Fairburn

**Affiliations:** a Department of Eating and Weight Disorders, Villa Garda Hospital, Via Montebaldo, 89, I-37016 Garda, VR, Italy; b Oxford University, Department of Psychiatry, Warneford Hospital, Oxford OX3 7JX, UK

**Keywords:** Eating disorders, Treatment, Cognitive behaviour therapy, Bulimia nervosa, Binge eating disorders, Adolescents

## Abstract

Little is known about the treatment of adolescents with an eating disorder who are not underweight. Enhanced cognitive behaviour therapy (CBT-E) is a potential option as it is a treatment for adult patients with eating disorders of this type and it has been shown to be effective with adolescent patients who are underweight. The aim of the present cohort study was to evaluate the effects of CBT-E on non-underweight adolescents with an eating disorder. Sixty-eight adolescent patients with an eating disorder and a body mass index (BMI) centile corresponding to an adult BMI ≥18.5 were recruited from consecutive referrals to a community-based eating disorder clinic. Each was offered 20 sessions of CBT-E over 20 weeks. Three-quarters completed the full 20 sessions. There was a marked treatment response with two-thirds (67.6%, intent-to-treat) having minimal residual eating disorder psychopathology by the end of treatment. CBT-E therefore appears to be a promising treatment for those adolescents with an eating disorder who are not underweight.

## Highlights

• A cohort of non-underweight adolescents with an eating disorder was treated with enhanced CBT (CBT-E).
• Three-quarters completed the full 20 sessions.
• Two-thirds had minimal eating disorder psychopathology by the end (intent-to-treat).
• CBT-E appears to be a promising treatment for this patient group.

## 1 Introduction

There are now substantial data supporting the use of “enhanced cognitive behaviour therapy” (CBT-E) in the treatment of adults with an eating disorder. The main studies have been transdiagnostic in their recruitment and they fall into two groups. One group has focused on adult patients who are not underweight (Byrne, Fursland, Allen, & Watson, 2011; Fairburn et al., 2015; Fairburn et al., 2009) whereas the other has focused on adults who are underweight (Fairburn et al., 2013). There have also been studies of patients meeting diagnostic criteria for a specific eating disorder (Poulsen et al., 2014).

There has been much less research on the use of CBT-E with adolescent patients. There have been just two studies and both have focused on patients who are underweight. The first study assessed the immediate and longer-term effects of CBT-E in a cohort of adolescent outpatients with anorexia nervosa (Dalle Grave, Calugi, Doll, & Fairburn, 2013). Two-thirds completed treatment. In these patients there was a substantial increase in weight accompanied by a marked decrease in eating disorder psychopathology that was well maintained over a 60-week period of follow-up. The second study was of adolescent inpatients with severe anorexia nervosa (Dalle Grave, Calugi, El Ghoch, Conti, & Fairburn, 2014). The great majority completed the CBT-E-based treatment programme. Most had a good outcome and there was a low rate of relapse following discharge.

The aim of the present study was to evaluate the effects of CBT-E in a cohort of adolescents who were not underweight (i.e., those with bulimia nervosa, binge eating disorder or one of the other non-underweight eating disorder presentations).

## 2 Method

### 2.1 Design

A cohort of non-underweight adolescent patients with an eating disorder was recruited from consecutive referrals to a community-based eating disorder clinic. Eligible patients were offered 20 sessions of CBT-E over 20 weeks. This was their sole psychological intervention. The study was reviewed and approved by the Institutional Review Board of Villa Garda Hospital, Verona. The goal was to recruit 60 patients so that the study had 95% power to detect a moderate change on EDE-Q global score from baseline equivalent to an effect size of around 0.5.

### 2.2 Setting and participants

The sample was recruited from consecutive referrals by family doctors and other clinicians to a well-established eating disorder clinic serving the Verona area of Italy. To be eligible, patients had to be aged between 13 and 19 years with a BMI centile corresponding to an adult BMI ≥18.5 and to fulfil the DSM-IV diagnostic criteria for bulimia nervosa or eating disorder not otherwise specified (American Psychiatric Association, 1994). The exclusion criteria were as follows: i) prior receipt of a treatment closely resembling CBT-E (N = 3); having a co-existing Axis 1 psychiatric disorder that precluded immediate eating disorder-focused treatment (e.g., substance use disorders, acute psychotic disorders, high suicidal risk) (N = 13); iii) not being available for the 20 week period of treatment (N = 4); and (iv) having a medical instability or pregnancy (N = 0).

### 2.3 The treatment

CBT-E is a treatment for patients with eating disorder psychopathology, irrespective of their eating disorder diagnosis. With non-underweight adults it involves an initial assessment appointment followed by 20 treatment sessions over 20 weeks. The strategies and procedures used in CBT-E are described in a detailed treatment guide (Fairburn, 2008).

In this study of adolescents the same protocol was used except that the patients' parents were involved given the patients' age and circumstances. The parental involvement was peripheral and consisted of a single assessment session during the first two weeks plus four brief sessions with the patient and parents together immediately after an individual session with the patient (at weeks 4, 8, 12 and 20). The aim of the initial session was to identify and address family factors liable to hinder the patient's attempts to change while the subsequent sessions were devoted to the generation of solutions to problems implementing CBT-E. There were additional sessions with the parents if there were family crises, extreme difficulties at mealtimes or parental hostility towards the adolescent.

A single therapist treated each patient with a substitute stepping in if the primary therapist had to be absent. There was no additional therapeutic input, either from physicians, dieticians or other health professionals other than an initial assessment by a physician (RDG) to check that the patient was suitable for outpatient treatment and reassessment if there were physical concerns (e.g., due to weight loss or frequent purging).

Three clinical psychologists delivered the treatment. All had generic clinical experience and experience treating patients with eating disorders. Each therapist received six months' initial training from RDG and CGF. Weekly supervision meetings were led by RDG. The therapists also had six-monthly booster workshops led by CGF. All the sessions were recorded and these recordings were used as part of supervision to ensure that the treatment was well implemented.

### 2.4 Assessment

There were two assessment points; before treatment, and at the end of treatment.

*Body weight and body mass index*. Weight was measured using a beam balance scale and height was measured using a wall-mounted stadiometer. Body mass index (BMI) centiles were calculated using the Center for Disease Control and Prevention growth charts (www.cdc.gov/growthcharts). A BMI centile corresponding to an adult BMI ≥18.5 was calculated following the procedure described by Cole, Flegal, Nicholls, and Jackson (2007).

*Eating disorder features*. The Italian version of the self-report Eating Disorder Examination Questionnaire (EDE-Q6.0) was used (Fairburn & Beglin, 2008).

*General psychiatric features*. The full version of the Symptom Checklist-90 was used from which a Global Severity Index (GSI) was calculated (Derogatis, 1977).

### 2.5 Statistical analysis

The primary analysis was an intent-to-treat analysis. It was performed by replacing missing end of treatment data with baseline data. The analyses was undertaken by SC using standard treatment research data analytic procedures. Data are presented as N (%) for categorical data and as means (with standard deviation, SD) or medians (with range) for continuous data. T-test or Mann–Whitney and Chi-squared test were used to compare continuous and categorical measures, between two groups (completers and non-completers), as appropriate. McNemar tests for categorical data and paired t-tests or Wilcoxon test (as appropriate) for continuous data were used to compare differences within groups. Effect size (Cohen's d and *r*, as appropriate) was calculated to assess the magnitude of any differences between the groups.

## 3 Results

### 3.1 Sample

Sixty-eight patients were recruited, of whom 20 (29.4%) had bulimia nervosa, 14 (20.6%) had binge eating disorder, and the remaining 34 (50.0%) had another presentation. The mean age was 16.5 years (SD 1.7; range 13–19 years). All the patients were single and living with their family of origin. Two were male. The mean duration of eating disorder was 1.7 years (SD 1.8; range 0–6, median 1 year).

### 3.2 Treatment completion

Three quarters of the patients (51/68; 75.0%) completed the full 20 sessions of CBT-E. The completion rates by diagnosis were as follows: bulimia nervosa – 65.0% (13/20); binge eating disorder - 71.4% (10/14); other eating disorder – 82.3% (28/34). The completers and non-completers had similar baseline characteristics, except for weight and BMI centile which were significantly lower in the completers than the non-completers (weight: 54.9 ± 8.1 vs 59.6 ± 8.7, p = 0.027; BMI centile: 39.3 ± 22.1 vs 51.6 ± 24.8, p = 0.034; respectively).

Little additional therapeutic input was needed. Eight patients had between two to four extra parental sessions, and five patients were reassessed by a physician because of physical concerns.

### 3.3 Response to treatment (intent-to-treat)

There was a marked response to treatment across all measures (see Table 1). By the end of treatment the mean intent-to-treat global EDE-Q score decreased from 3.6 (SD 1.5) to 1.8 (SD 1.8, d = 1.03), and the mean GSI from 1.4 (SD 0.7) to 0.9 (SD 0.8, d = 0.66). Forty-six patients (67.6%) had minimal residual eating disorder psychopathology, defined as having a global EDE-Q score below 1 SD above the community mean (Mond, Hay, Rodgers, & Owen, 2006) (i.e., <2.77). The frequency of binge eating, self-induced vomiting and laxative misuse decreased substantially. Of those who were binge eating or purging at the beginning of treatment, half (25/50, 50%) had ceased all these forms of behaviour by the end. Body weight increased slightly from 56.1 kg (SD 8.3) to 57.1 kg (SD 7.2; p = 0.039, d = −0.14).

### 3.4 Response among treatment completers

The response of those who completed treatment was substantial (see Table 2). The mean global EDE-Q score decreased by 2.1 (SD 1.5; 95% CI 1.7–2.6; p < 0.001) and the mean GSI decreased by 0.9 (SD 0.9; 95% CI 0.6–1.1; p < 0.001). By the end of treatment over eighty percent (42/51; 82.4%) met criteria for having minimal residual eating disorder psychopathology, and of those who were binge eating or purging at the beginning of treatment, 76.5% (26/34) had ceased all these forms of behaviour by the end.

## 4 Discussion

The aim of the present study was to determine whether CBT-E might be a potential treatment for adolescent patients with an eating disorder who are not underweight. To achieve this aim, eligible referrals to a community-based eating disorder clinic were treated with 20 sessions of CBT-E over 20 weeks.

There were two main findings. The first was that three-quarters of the patients completed the full 20 sessions of CBT-E. Few had any additional therapeutic input. The second finding was that there were substantial improvements in eating disorder psychopathology and general psychiatric features. Two-thirds of those who started treatment (67.6%) had minimal residual eating disorder psychopathology by the end, and half of those who had been binge eating and purging had ceased both forms of behaviour (intent-to-treat figures).

The study had certain strengths. First, it assessed for the first time the effects of CBT-E in non-underweight adolescent patients. Second, the sample was a clinically relevant one as it was recruited from consecutive referrals to a catchment area eating disorder clinic with few exclusion criteria being applied. The findings are therefore likely to be generalizable to mainstream specialist services. Third, the study had good internal validity as the therapists were well trained and closely supervised. The study had one main limitation. This was that there was no follow-up so it is not possible to say whether the treatment effects persisted.

A potential threat to the generalisability of the findings is the fact that the therapists were well trained. This problem may be more apparent than real since data from three “real world” clinics indicate that the outcome of patients who complete CBT-E, or a CBT-E-like treatment, is similar to that obtained in the main randomised controlled trials although the completion rates are lower (Byrne et al., 2011; Knott, Woodward, Hoefkens, & Limbert, 2014; Turner, Marshall, Stopa, & Waller, 2015). Nevertheless the scaling up of therapist training is a challenge. One possible solution is “web-centred training” (Fairburn & Cooper, 2011; Fairburn & Patel, 2014). It centres on the use of a specially designed website which describes and illustrates the treatment in great detail and incorporates tasks to help trainees grasp key concepts and master the main procedures. Two such websites have been developed, one of which focuses on CBT-E. Over four hundred therapists have embarked upon this training and its effectiveness is under evaluation.

Also of relevance to the present study are the two studies of the treatment of bulimia nervosa in adolescents, one comparing family therapy with supportive psychotherapy (Le Grange, Crosby, Rathouz, & Leventhal, 2007) and the other comparing family therapy with guided self-help (Schmidt et al., 2007). There have been no studies of the treatment of binge eating disorder or the other eating disorder presentations yet they form the majority of cases (48 out of the 68 cases in the present cohort; 70.6%) (Ornstein et al., 2013; Smink, van Hoeken, Oldehinkel, & Hoek, 2014).

In light of the present findings CBT-E appears to be a promising treatment for non-underweight adolescents with an eating disorder. It may be a particularly attractive treatment as it can be used with all the presentations encountered, whether the patient is underweight (Dalle Grave et al., 2013, 2014) or not. The obvious next research step is to compare CBT-E with the leading other approach for adolescents, family-based treatment, and to do so across the full range of eating disorder presentations.

## Figures and Tables

**Table 1:** Characteristics of the patients before and after treatment (intent to treat data set, n = 68).

	Before treatment	After treatment			
	Mean (SD)	Mean (SD)	T-Test	p	Cohen's d
**Weight**					
Body weight (kg)	56.1 (8.3)	57.1 (7.2)	−2.10	0.039	−0.14
Body mass index centile	42.9 (23.2)	46.5 (21.7)	−2.00	0.051	−0.16
**Eating disorder psychopathology**					
Overall severity (global EDE-Q)	3.6 (1.5)	1.8 (1.8)	8.33	<0.001	1.03
Dietary restraint (EDE-Q subscale)	3.6 (1.7)	1.6 (1.8)	8.36	<0.001	1.14
Eating concern (EDE-Q subscale)	3.2 (1.4)	1.5 (1.7)	8.09	<0.001	1.09
Weight concern (EDE-Q subscale)	3.5 (1.8)	1.8 (1.9)	7.11	<0.001	0.92
Shape concern (EDE-Q subscale)	4.0 (1.8)	2.2 (2.0)	7.43	<0.001	0.95
**General psychiatric features, GSI**	1.4 (0.7)	0.9 (0.8)	6.27	<0.001	0.66
	N (%)	N (%)	McNemar test or Fisher exact test	p	
Global EDE-Q <1 SD above the community mean^a^	17 (25.0)	46 (67.6)	27.03	0.001	
**Eating disorder behaviour (EDE-Q)**					
Objective bulimic episodes ≥1	37 (54.4)	17 (25.0)		<0.001	
Self-induced vomiting ≥1	27 (39.7)	12 (17.6)		<0.001	
Laxative taking ≥1	9 (13.2)	5 (7.4)		0.289	
Absence of all of the above	18 (26.5)	41 (60.3)	17.93	<0.001	
Cessation of all of the above, if present at baseline	–	25/50 (50.0%)	–	–	
	Median (range)	Median (range)	Wilcoxon test	p	*r*
Objective bulimic episodes (N)	1 (0–84)	0 (0–35)	−3.98	<0.001	0.48
Self-induced vomiting (N)	0 (0–80)	0 (0–65)	−3.63	0.001	0.44
Laxative taking (N)	0 (0–30)	0 (0–15)	−1.40	0.161	0.17

EDE-Q – Eating Disorder Examination-Questionnaire (version 6.0); GSI – Global severity index.

a Global EDE-Q less than 1 SD above community EDE-Q mean for young adult women (i.e., below 2.77).

**Table 2:** Characteristics of the patients before and after treatment among those who completed treatment (n = 51).

	Before treatment	After treatment			
	Mean (SD)	Mean (SD)	T-Test	p	Cohen's d
**Weight**					
Body weight (kg)	54.9 (8.1)	56.4 (6.8)	−2.35	0.039	−0.20
Body mass index centile	39.3 (22.1)	44.1 (20.7)	−2.23	0.051	−0.22
**Eating disorder psychopathology**					
Overall severity (global EDE-Q)	3.5 (1.4)	1.2 (1.4)	9.81	<0.001	1.64
Dietary restraint (EDE-Q subscale)	3.6 (1.6)	1.0 (1.4)	10.09	<0.001	1.73
Eating concern (EDE-Q subscale)	3.2 (1.4)	0.9 (1.3)	9.51	<0.001	1.70
Weight concern (EDE-Q subscale)	3.5 (1.7)	1.3 (1.5)	7.95	<0.001	1.37
Shape concern (EDE-Q subscale)	4.0 (1.8)	1.7 (1.7)	8.25	<0.001	1.31
**General psychiatric features, GSI**	1.4 (0.8)	0.6 (0.7)	6.60	<0.001	1.13
	N (%)	N (%)	McNemar test or Fisher exact test	p	
Global EDE-Q <1 SD above the community mean^a^	13 (25.5)	42 (82.4)	27.03	<0.001	
**Eating disorder behaviour (EDE-Q)**					
Objective bulimic episodes ≥1	27 (52.9)	6 (11.8)		<0.001	
Self-induced vomiting ≥1	19 (37.3)	3 (5.9)		<0.001	
Laxative taking ≥1	7 (13.7)	2 (3.9)		0.289	
Absence of all of the above	17 (33.3)	40 (83.3)	17.93	<0.001	
Cessation of all of the above, if present at baseline	–	26/34 (76.5)	–	–	
	Median (range)	Median (range)	Wilcoxon test	p	*r*
Objective bulimic episodes (N)	1 (0–84)	0 (0–6)	−3.98	<0.001	0.56
Self-induced vomiting (N)	0 (0–80)	0 (0–4)	−3.63	0.001	0.51
Laxative taking (N)	0 (0–30)	0 (0–15)	−1.40	0.161	0.20

EDE-Q – Eating Disorder Examination-Questionnaire (version 6.0); GSI – Global severity index.

Missing values: at end of treatment there were a further 2 missing values for BMI and 3 missing values for EDE-Q and GSI.

a Global EDE-Q less than 1 SD above community EDE-Q mean for young adult women (i.e., below 2.77).
